# 1,6-Bis[(2,2′:6′,2′′-terpyridin-4′-yl)­oxy]hexa­ne

**DOI:** 10.1107/S1600536812029017

**Published:** 2012-06-30

**Authors:** Varvara I. Nikolayenko, Matthew P. Akerman, Craig D. Grimmer, Desigan Reddy

**Affiliations:** a University of KwaZulu-Natal, School of Chemistry and Physics, Private Bag X01, Scottsville 3209, Pietermaritzburg, South Africa

## Abstract

The mol­ecule of the title compound, C_36_H_32_N_6_O_2_, lies about an inversion center, located at the mid-point of the central C—C bond of the diether bridge. The terminal pyridine rings form dihedral angles of 4.67 (7) and 26.23 (7)° with the central ring. In the crystal, weak C—H⋯N and C—H⋯O inter­actions link the mol­ecules into a three-dimensional network.

## Related literature

For the structure of the unsubstituted 2,2′:6′,2"-terpyridine, see: Bessel *et al.* (1992 ▶). For the structure of the precursor to the title compound, 4′-chloro-2,2′:6′,2"-terpyridine, see: Beves *et al.* (2006 ▶). For the structure of the 1,4-bis­[(2,2′:6′,2"-terpyridin-4′-yl)­oxy]-butane, see: Akerman *et al.* (2011 ▶). For a full review of functionalized 2,2′:6′,2"-terpyridine complexes, see: Fallahpour (2003 ▶); Heller & Schubert (2003 ▶). For a comprehensive summary of platinum(II) terpyridine complexes, see: Newkome *et al.* (2008 ▶). For the structure of bis­(2,2′:6′,2"-terpyrid­yl)ether, see: Constable *et al.* (1995 ▶). For the structure of related bis­(terpyridine) compounds, linked by an alk­oxy spacer, see: Constable *et al.* (2006 ▶). For the synthetic procedure, see: Constable *et al.* (2005 ▶); Van der Schilden (2006 ▶).
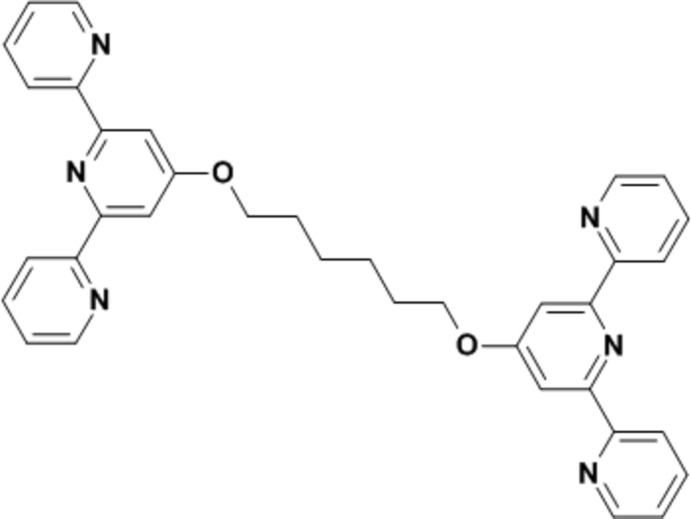


## Experimental

### 

#### Crystal data

C_36_H_32_N_6_O_2_
*M* *_r_* = 580.7
Orthorhombic, 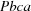

*a* = 15.139 (5) Å
*b* = 11.428 (5) Å
*c* = 16.760 (5) Å
*V* = 2899.6 (18) Å^3^
*Z* = 4
Mo *K*α radiation
μ = 0.09 mm^−1^
*T* = 100 K
0.40 × 0.20 × 0.20 mm

#### Data collection

Oxford Diffraction Xcalibur 2 CCD diffractometer
Absorption correction: multi-scan (Blessing, 1995 ▶) *T*_min_ = 0.967, *T*_max_ = 0.983
20354 measured reflections
2859 independent reflections
2098 reflections with *I* > 2σ(*I*)
*R*_int_ = 0.064

#### Refinement

*R*[*F* ^2^ > 2σ(*F* ^2^)] = 0.038
*wR*(*F* ^2^) = 0.100
*S* = 0.94
2859 reflections
199 parameters
H-atom parameters constrained
Δρ_max_ = 0.17 e Å^−3^
Δρ_min_ = −0.26 e Å^−3^

### 

Data collection: *CrysAlis CCD* (Oxford Diffraction, 2008 ▶); cell refinement: *CrysAlis CCD*; data reduction: *CrysAlis RED* (Oxford Diffraction, 2008 ▶); program(s) used to solve structure: *SHELXS97* (Sheldrick, 2008 ▶); program(s) used to refine structure: *SHELXL97* (Sheldrick, 2008 ▶); molecular graphics: *ORTEP-3* (Farrugia, 1999 ▶); software used to prepare material for publication: *publCIF* (Westrip, 2010 ▶).

## Supplementary Material

Crystal structure: contains datablock(s) I, global. DOI: 10.1107/S1600536812029017/yk2063sup1.cif

Structure factors: contains datablock(s) I. DOI: 10.1107/S1600536812029017/yk2063Isup2.hkl

Supplementary material file. DOI: 10.1107/S1600536812029017/yk2063Isup3.cml

Additional supplementary materials:  crystallographic information; 3D view; checkCIF report

## Figures and Tables

**Table 1:** Short intermolecular contacts (Å, °)

*D*—H⋯*A*	*D*—H	H⋯*A*	*D*⋯*A*	*D*—H⋯*A*
C15—H15⋯O^i^	0.95	2.65	3.575 (2)	164
C1—H1⋯N3^ii^	0.95	2.71	3.654 (2)	174
C4—H4⋯N1^iii^	0.95	2.65	3.402 (2)	136
C2—H2⋯O^ii^	0.95	2.69	3.627 (2)	168

Symmetry codes: (i) 
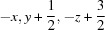
; (ii) 
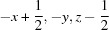
; (iii) 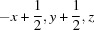.

**Fig. 1.**
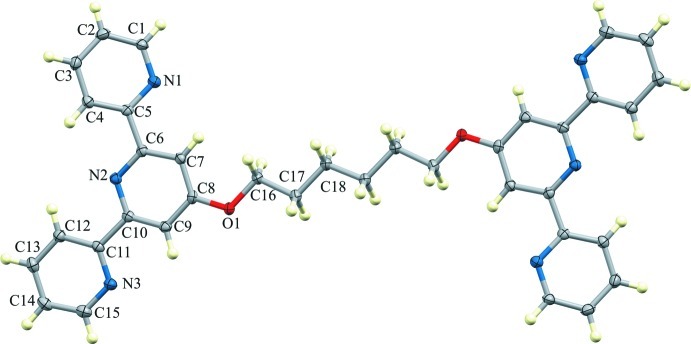
The molecular structure of the title compound. Displacement ellipsoids are drawn at the 50% probability level.

**Fig. 2.**
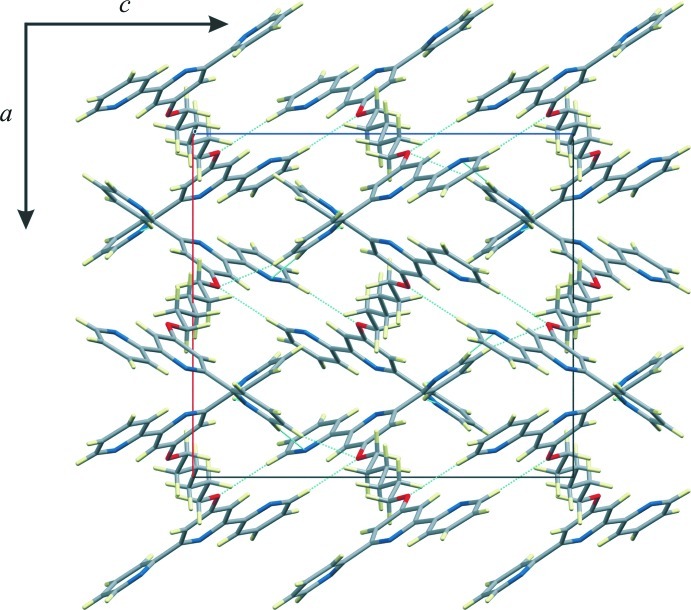
Intermolecular C—H···O and C—H···N contacts responsible for the three-dimensional network in crystals of the title compound, viewed down the b axis.

## supplementary crystallographic information

### Comment

The title compound is the second in a series of ligands developed in an effort to harness multifunctional activity. Coordination of these ligands to platinum(II) should enable covalent binding of DNA through both metal centres, thus increasing the number of adducts formed. Furthermore the presence of the flexible diol derived linkage will provide the complex with the potential to engage in long range interactions with DNA.

The ligand crystallized in the orthorhombic space group Pbca, with a half molecule in the asymmetric unit and *Z* = 4. Crystallographically imposed inversion symmetry relates two halves of the ligand. The inversion center is located at the mid-point of the diol linkage. The three pyridine rings adopt a *trans, trans* conformation. The same configuration is observed in the parent 4'-chloro-2,2': 6',2''- terpyridine (Beves *et al.*, 2006) and is a common feature of uncoordinated terpyridine ligands in general (Akerman *et al.*, 2011; Bessel *et al.*, 1992).

The central pyridine ring of the terpyridine fragment lie in the same plane as the bridging chain. The terminal pyridine rings are, however, canted relative to the central ring. The C7–C6–C5–N1 torsion angle is -25.8 (2)°, while the C9–C10–C11–N3 torsion angle is 4.9 (2)° (Fig. 1). The large torsion angle formed by one of terminal pyridine groups with the central ring is seemingly to allow for interaction between the pyridine N1 atom and the hydrogen atom H4 of an adjacent molecule, with the distance of 2.65 Å. There are also other short contacts C—H···O and C—H···N, ranging from 2.65 to 2.71 Å. These contacts link the molecules into a herringbone pattern (Figure 2). There is no indication of meaningful π··· π or C–H··· π interactions in the lattice, which are often observed in terpyridine structures (Beves *et al.* 2006).

### Experimental

The title compound was prepared by an adaptation of a previously described method (Van der Schilden, 2006; Constable *et al.*, 2005). Hexanediol (1.13 mmol) was added to a suspension of ground potassium hydroxide (6.69 mmol) in DMSO (30 ml). The solution was heated to reflux for 1 h after which 4'-chloro-2,2':6',2''-terpyridine (2.23 mmol) was added. The mixture was again brought to reflux for an additional 24 h. After cooling to room temperature, the brown mixture was added to cold water (100 ml). The resulting off-white precipitate was filtered, rinsed with cold ethanol and air dried. Single crystals were grown by slow liquid diffusion of *n*-hexane into a chloroform solution of the compound.

### Refinement

All non-hydrogen atoms were located in the difference Fourier map and refined anisotropically. The positions of all hydrogen atoms were calculated using the riding model with C—H(aromatic) and C—H(methylene) distances of 0.93 Å and *U*_iso_ = 1.2 *U*_eq_.

### Crystal data

**Table:**

C_36_H_32_N_6_O_2_	*F*(000) = 1224
*M**_r_* = 580.7	*D*_x_ = 1.330 Mg m^−^^3^
Orthorhombic, *P**b**c**a*	Mo *K*α radiation, λ = 0.71073 Å
Hall symbol: -P 2ac 2ab	Cell parameters from 2098 reflections
*a* = 15.139 (5) Å	θ = 3.2–26.0°
*b* = 11.428 (5) Å	µ = 0.09 mm^−^^1^
*c* = 16.760 (5) Å	*T* = 100 K
*V* = 2899.6 (18) Å^3^	Needle, colourless
*Z* = 4	0.40 × 0.20 × 0.20 mm

### Data collection

**Table:**

Oxford Diffraction Xcalibur 2 CCD diffractometer	2859 independent reflections
Radiation source: fine-focus sealed tube	2098 reflections with *I* > 2σ(*I*)
Graphite monochromator	*R*_int_ = 0.064
ω scans at fixed θ angles	θ_max_ = 26.1°, θ_min_ = 3.2°
Absorption correction: multi-scan (Blessing, 1995)	*h* = −18→18
*T*_min_ = 0.967, *T*_max_ = 0.983	*k* = −12→14
20354 measured reflections	*l* = −20→20

### Refinement

**Table:**

Refinement on *F*^2^	Primary atom site location: structure-invariant direct methods
Least-squares matrix: full	Secondary atom site location: difference Fourier map
*R*[*F*^2^ > 2σ(*F*^2^)] = 0.038	Hydrogen site location: inferred from neighbouring sites
*wR*(*F*^2^) = 0.100	H-atom parameters constrained
*S* = 0.94	*w* = 1/[σ^2^(*F*_o_^2^) + (0.0644*P*)^2^ + 0.*P*] where *P* = (*F*_o_^2^ + 2*F*_c_^2^)/3
2859 reflections	(Δ/σ)_max_ = 0.001
199 parameters	Δρ_max_ = 0.17 e Å^−^^3^
0 restraints	Δρ_min_ = −0.26 e Å^−^^3^

### Special details

**Table:**

Geometry. All s.u.'s (except the s.u. in the dihedral angle between two l.s. planes) are estimated using the full covariance matrix. The cell s.u.'s are taken into account individually in the estimation of s.u.'s in distances, angles and torsion angles; correlations between s.u.'s in cell parameters are only used when they are defined by crystal symmetry. An approximate (isotropic) treatment of cell s.u.'s is used for estimating s.u.'s involving l.s. planes.
Refinement. Refinement of *F*^2^ against ALL reflections. The weighted *R*-factor *wR* and goodness of fit *S* are based on *F*^2^, conventional *R*-factors *R* are based on *F*, with *F* set to zero for negative *F*^2^. The threshold expression of *F*^2^ > σ(*F*^2^) is used only for calculating *R*-factors(gt) *etc*. and is not relevant to the choice of reflections for refinement. *R*-factors based on *F*^2^ are statistically about twice as large as those based on *F*, and *R*- factors based on ALL data will be even larger.

### Fractional atomic coordinates and isotropic or equivalent isotropic displacement parameters (Å^2^)

**Table:**

	*x*	*y*	*z*	*U*_iso_*/*U*_eq_	
C1	0.33229 (9)	0.02089 (12)	0.29739 (9)	0.0207 (3)	
H1	0.3600	−0.0452	0.2738	0.025*	
C2	0.34439 (10)	0.13004 (12)	0.26216 (9)	0.0251 (4)	
H2	0.3790	0.1379	0.2152	0.030*	
C3	0.30541 (10)	0.22649 (12)	0.29648 (9)	0.0242 (3)	
H3	0.3124	0.3019	0.2734	0.029*	
C4	0.25569 (9)	0.21180 (12)	0.36541 (8)	0.0203 (3)	
H4	0.2288	0.2770	0.3907	0.024*	
C5	0.24615 (9)	0.10061 (12)	0.39646 (8)	0.0168 (3)	
C6	0.19174 (9)	0.07961 (12)	0.46867 (8)	0.0165 (3)	
C7	0.15172 (9)	−0.02860 (12)	0.48099 (8)	0.0176 (3)	
H7	0.1608	−0.0915	0.4448	0.021*	
C8	0.09834 (9)	−0.04198 (12)	0.54744 (8)	0.0171 (3)	
C9	0.08764 (9)	0.05263 (12)	0.59918 (8)	0.0181 (3)	
H9	0.0506	0.0462	0.6447	0.022*	
C10	0.13161 (9)	0.15523 (12)	0.58311 (8)	0.0169 (3)	
C11	0.12409 (9)	0.25867 (12)	0.63712 (8)	0.0183 (3)	
C12	0.16340 (10)	0.36471 (12)	0.61690 (9)	0.0210 (3)	
H12	0.1964	0.3725	0.5690	0.025*	
C13	0.15333 (10)	0.45889 (13)	0.66836 (9)	0.0249 (4)	
H13	0.1788	0.5327	0.6559	0.030*	
C14	0.10571 (10)	0.44385 (13)	0.73784 (9)	0.0251 (4)	
H14	0.0972	0.5071	0.7738	0.030*	
C15	0.07083 (10)	0.33492 (13)	0.75376 (9)	0.0248 (4)	
H15	0.0398	0.3245	0.8025	0.030*	
C16	0.06992 (9)	−0.24153 (11)	0.51510 (9)	0.0194 (3)	
H16A	0.1330	−0.2642	0.5175	0.023*	
H16B	0.0554	−0.2212	0.4592	0.023*	
C17	0.01309 (10)	−0.34175 (11)	0.54245 (9)	0.0197 (3)	
H17A	−0.0499	−0.3192	0.5396	0.024*	
H17B	0.0272	−0.3612	0.5986	0.024*	
C18	0.02948 (9)	−0.44830 (11)	0.48956 (9)	0.0194 (3)	
H18A	0.0192	−0.4263	0.4332	0.023*	
H18B	0.0920	−0.4722	0.4948	0.023*	
N1	0.28336 (8)	0.00494 (10)	0.36295 (7)	0.0196 (3)	
N2	0.18298 (8)	0.17077 (9)	0.51806 (7)	0.0176 (3)	
N3	0.07781 (8)	0.24306 (10)	0.70493 (7)	0.0205 (3)	
O	0.05447 (6)	−0.14203 (8)	0.56613 (6)	0.0204 (2)	

### Atomic displacement parameters (Å^2^)

**Table:**

	*U*^11^	*U*^22^	*U*^33^	*U*^12^	*U*^13^	*U*^23^
C1	0.0226 (8)	0.0216 (8)	0.0178 (7)	−0.0008 (6)	0.0034 (6)	−0.0049 (6)
C2	0.0274 (9)	0.0297 (9)	0.0183 (8)	−0.0054 (6)	0.0059 (6)	−0.0001 (7)
C3	0.0310 (8)	0.0187 (7)	0.0230 (8)	−0.0061 (6)	0.0010 (7)	0.0019 (6)
C4	0.0230 (8)	0.0180 (7)	0.0198 (7)	−0.0019 (6)	−0.0002 (6)	−0.0025 (6)
C5	0.0176 (7)	0.0190 (7)	0.0137 (7)	−0.0016 (6)	−0.0026 (6)	−0.0010 (6)
C6	0.0175 (7)	0.0164 (7)	0.0155 (7)	0.0008 (5)	−0.0018 (6)	0.0011 (6)
C7	0.0202 (8)	0.0157 (7)	0.0168 (7)	0.0005 (5)	−0.0006 (6)	−0.0014 (6)
C8	0.0175 (7)	0.0149 (7)	0.0189 (7)	−0.0013 (5)	−0.0029 (6)	0.0024 (6)
C9	0.0203 (8)	0.0199 (7)	0.0141 (7)	0.0011 (6)	−0.0001 (6)	0.0000 (6)
C10	0.0173 (7)	0.0184 (7)	0.0150 (7)	0.0019 (5)	−0.0017 (6)	0.0000 (6)
C11	0.0185 (7)	0.0194 (7)	0.0172 (7)	0.0015 (6)	−0.0016 (6)	−0.0023 (6)
C12	0.0228 (8)	0.0210 (8)	0.0191 (8)	−0.0016 (6)	0.0006 (6)	−0.0015 (6)
C13	0.0268 (8)	0.0179 (8)	0.0299 (8)	−0.0012 (6)	−0.0026 (7)	−0.0035 (6)
C14	0.0265 (8)	0.0243 (8)	0.0246 (8)	0.0020 (6)	−0.0016 (7)	−0.0090 (7)
C15	0.0251 (8)	0.0284 (8)	0.0209 (8)	0.0028 (7)	0.0015 (7)	−0.0047 (7)
C16	0.0237 (7)	0.0146 (7)	0.0198 (7)	−0.0008 (6)	0.0020 (6)	−0.0018 (6)
C17	0.0228 (8)	0.0164 (7)	0.0199 (8)	−0.0006 (6)	0.0014 (6)	−0.0002 (6)
C18	0.0212 (8)	0.0163 (7)	0.0208 (7)	−0.0008 (6)	0.0023 (6)	−0.0004 (6)
N1	0.0215 (6)	0.0190 (6)	0.0184 (6)	0.0003 (5)	0.0008 (5)	−0.0016 (5)
N2	0.0205 (6)	0.0165 (6)	0.0159 (6)	0.0007 (5)	−0.0010 (5)	−0.0004 (5)
N3	0.0226 (6)	0.0214 (6)	0.0175 (6)	0.0001 (5)	0.0023 (5)	−0.0025 (5)
O	0.0262 (6)	0.0146 (5)	0.0203 (5)	−0.0041 (4)	0.0044 (4)	−0.0012 (4)

### Geometric parameters (Å, º)

**Table:**

C1—N1	1.3374 (18)	C10—N2	1.3508 (18)
C1—C2	1.392 (2)	C10—C11	1.4934 (19)
C2—C3	1.377 (2)	C11—N3	1.3467 (18)
C3—C4	1.389 (2)	C11—C12	1.391 (2)
C4—C5	1.380 (2)	C12—C13	1.388 (2)
C5—N1	1.3527 (18)	C13—C14	1.380 (2)
C5—C6	1.4833 (19)	C14—C15	1.378 (2)
C6—N2	1.3374 (18)	C15—N3	1.3354 (18)
C6—C7	1.3917 (19)	C16—O	1.4422 (17)
C7—C8	1.3845 (19)	C16—C17	1.5031 (19)
C8—O	1.3581 (17)	C17—C18	1.527 (2)
C8—C9	1.395 (2)	C18—C18^i^	1.521 (3)
C9—C10	1.374 (2)		
			
N1—C1—C2	122.88 (13)	N2—C10—C11	115.45 (12)
C3—C2—C1	118.96 (14)	C9—C10—C11	121.29 (12)
C2—C3—C4	118.91 (13)	N3—C11—C12	122.87 (13)
C5—C4—C3	118.73 (13)	N3—C11—C10	116.54 (12)
N1—C5—C4	122.98 (13)	C12—C11—C10	120.59 (13)
N1—C5—C6	116.09 (12)	C13—C12—C11	118.48 (14)
C4—C5—C6	120.91 (12)	C14—C13—C12	119.06 (14)
N2—C6—C7	123.83 (13)	C15—C14—C13	118.38 (14)
N2—C6—C5	115.77 (12)	N3—C15—C14	124.12 (14)
C7—C6—C5	120.39 (12)	O—C16—C17	109.06 (11)
C8—C7—C6	118.09 (13)	C16—C17—C18	109.71 (12)
O—C8—C7	124.28 (12)	C18^i^—C18—C17	112.98 (15)
O—C8—C9	116.87 (12)	C1—N1—C5	117.52 (12)
C7—C8—C9	118.86 (13)	C6—N2—C10	117.06 (11)
C10—C9—C8	118.87 (13)	C15—N3—C11	117.05 (12)
N2—C10—C9	123.26 (13)	C8—O—C16	116.58 (11)
			
N1—C1—C2—C3	0.8 (2)	N3—C11—C12—C13	−1.0 (2)
C1—C2—C3—C4	0.4 (2)	C10—C11—C12—C13	178.92 (12)
C2—C3—C4—C5	−0.9 (2)	C11—C12—C13—C14	0.7 (2)
C3—C4—C5—N1	0.3 (2)	C12—C13—C14—C15	0.7 (2)
C3—C4—C5—C6	−178.31 (13)	C13—C14—C15—N3	−2.1 (2)
N1—C5—C6—N2	155.72 (12)	O—C16—C17—C18	−179.36 (11)
C4—C5—C6—N2	−25.62 (19)	C16—C17—C18—C18^i^	−176.75 (15)
N1—C5—C6—C7	−25.77 (19)	C2—C1—N1—C5	−1.4 (2)
C4—C5—C6—C7	152.89 (13)	C4—C5—N1—C1	0.9 (2)
N2—C6—C7—C8	1.3 (2)	C6—C5—N1—C1	179.51 (12)
C5—C6—C7—C8	−177.08 (12)	C7—C6—N2—C10	−0.4 (2)
C6—C7—C8—O	179.13 (13)	C5—C6—N2—C10	178.08 (12)
C6—C7—C8—C9	−0.5 (2)	C9—C10—N2—C6	−1.4 (2)
O—C8—C9—C10	179.26 (12)	C11—C10—N2—C6	179.51 (12)
C7—C8—C9—C10	−1.1 (2)	C14—C15—N3—C11	1.8 (2)
C8—C9—C10—N2	2.1 (2)	C12—C11—N3—C15	−0.2 (2)
C8—C9—C10—C11	−178.83 (12)	C10—C11—N3—C15	179.86 (12)
N2—C10—C11—N3	−175.94 (12)	C7—C8—O—C16	3.91 (19)
C9—C10—C11—N3	4.92 (19)	C9—C8—O—C16	−176.42 (12)
N2—C10—C11—C12	4.17 (19)	C17—C16—O—C8	−177.42 (11)
C9—C10—C11—C12	−174.98 (14)		

Symmetry code: (i) −*x*, −*y*−1, −*z*+1.

### Hydrogen-bond geometry (Å, º)

**Table:**

*D*—H···*A*	*D*—H	H···*A*	*D*···*A*	*D*—H···*A*
C15—H15···O^ii^	0.95	2.65	3.575 (2)	164
C1—H1···N3^iii^	0.95	2.71	3.654 (2)	174
C4—H4···N1^iv^	0.95	2.65	3.402 (2)	136
C2—H2···O^iii^	0.95	2.69	3.627 (2)	168

Symmetry codes: (ii) −*x*, *y*+1/2, −*z*+3/2; (iii) −*x*+1/2, −*y*, *z*−1/2; (iv) −*x*+1/2, *y*+1/2, *z*.
